# Associations between gut microbiota and chronic sinusitis: A bidirectional Mendelian randomization study

**DOI:** 10.1002/iid3.1328

**Published:** 2024-07-19

**Authors:** Kunlin Pu, Zhipeng Zhang, Li Li

**Affiliations:** ^1^ Department of Otorhinolaryngology Pengzhou Hospital of Traditional Chinese Medicine Pengzhou China

**Keywords:** chronic sinusitis, gut microbiota, Mendelian randomization

## Abstract

**Background:**

Studies have indicated a close association between dysbiosis of the gut microbiota and chronic sinusitis. However, the causal relationship between the gut microbiota and the risk of chronic sinusitis remains unclear.

**Methods:**

Using genome‐wide association study (GWAS) data for the gut microbiota and chronic sinusitis, we conducted a two‐sample Mendelian randomization (MR) study to determine the potential causal relationship between the microbiota and chronic sinusitis. We employed the inverse variance‐weighted (IVW) method as the primary analytical approach to estimate the effect. Additionally, sensitivity, heterogeneity, and pleiotropy analyses were conducted to evaluate the robustness of the results. Reverse MR analysis was also applied to investigate potential reverse causality.

**Results:**

Through MR analysis, we identified 17 gut microbiota classifications that are closely associated with chronic sinusitis. However, after Bonferroni multiple correction, only class Bacilli (odds ratio: 0.785, 95% confidence interval: 0.677–0.911, *p* = .001, false discovery rate = 0.023) maintained a significant causal negative relationship with chronic sinusitis. Sensitivity analysis did not reveal any evidence of heterogeneity or horizontal pleiotropy. Reverse MR analysis found five gut microbiota classifications that are significantly associated with chronic sinusitis, but they were no longer significant after Bonferroni multiple correction. There was no evidence to suggest a reverse causal relationship between chronic sinusitis and class Bacilli.

**Conclusion:**

Specific gut microbiota predicted by genetics exhibit a potential causal relationship with chronic sinusitis, and class Bacilli may have a protective effect on chronic sinusitis.

## Graphical abstract

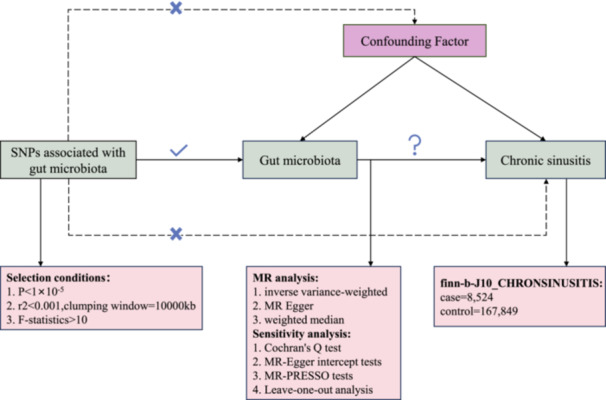

## 1 INTRODUCTION

Chronic sinusitis is characterized by chronic inflammation of the sinuses and nasal mucosa, with a duration of at least 12 weeks.^1^, ^2^ The prevalence of this disease varies from 5% to 14% worldwide, with rates of 10.9% in Europe and 11.9% in the United States.^3^, ^4^, ^5^, ^6^ This condition causes great distress to patients and increases the social burden. Increasing research focuses on potential risk factors associated with chronic sinusitis. Research has shown that individuals with select medical conditions, including asthma, chronic obstructive pulmonary disease (COPD), and gout, may have an increased risk of developing chronic sinusitis.^7^ Numerous studies have demonstrated a significant association between the incidence of sinusitis and inflammatory bowel disease.^8^, ^9^ The development of chronic sinusitis is influenced by various factors. In many cases of chronic sinusitis, viral infections are the main cause, while the role of bacteria in chronic sinusitis is still not well understood.^10^ Bacterial presence in the sinuses may exacerbate the symptoms of the disease. Additionally, dysbiosis of the microbial community in the nasal and sinus cavities is one of the mechanisms behind its occurrence.^11^ The gut microbiota has a close relationship with the nasal and sinus microbiota. Some studies suggest that individuals with chronic sinusitis may experience changes in their gut microbiota,^12^ but it is still uncertain whether the gut microbiota affects chronic sinusitis.

The human gastrointestinal tract harbors a complex and dynamic microbial community, including bacteria, archaea, and eukarya, collectively known as the “gut microbiota.”^13^ The gut microbiota is vital for preserving immune and metabolic homeostasis, while also providing defense against pathogens. Changes in gut microbiota composition are closely linked to the development and progression of numerous inflammatory diseases and infections.^14^, ^15^ The gut microbiota is critically important for various functions, such as extracting energy from the diet, producing vitamins and short‐chain fatty acids (SCFAs), and regulating immunity by modulating TH17 and Treg balance.^16^, ^17^ Additionally, the components released by the microbiota itself can impact the host, including lipopolysaccharides (LPS), cell membrane carbohydrates, and other endotoxins. These substances contribute to maintaining intestinal epithelial stability, generating critical immune system signaling molecules, and facilitating cellular interactions.^18^, ^19^ The nasal cavity is connected to the intestines through the pharynx and upper gastrointestinal tract, and alterations in the gut microbiota may affect the upper respiratory microbiota, thus being associated with the pathogenesis of chronic sinusitis. Studies have demonstrated dysbiosis in the gut microbiota of patients with chronic sinusitis, with decreased abundance of certain gut bacteria, leading to an imbalance between anti‐inflammatory and proinflammatory responses in the gut.^12^ This may be related to the inflammatory status of chronic sinusitis patients and suggests a potential link between gut microbiota dysbiosis and chronic sinusitis. However, the exact relationship between the two remains unclear.

Mendelian randomization (MR) uses genetic variation as an instrumental variable for the exposure factor to estimate the potential causal relationship between exposure and outcome.^20^ Due to the random allocation of genetic variations, MR can eliminate the influence of confounding factors and avoid reverse causation bias caused by allocating gene variations before the development of the disease. In this study, we conducted a bidirectional two‐sample MR analysis using summary statistics data from large‐scale genome‐wide association study (GWAS) on gut microbiota and chronic sinusitis to evaluate the potential causal relationship between gut microbiota and chronic sinusitis.

## 2 METHODS

### 2.1 Design

**Figure 1:**
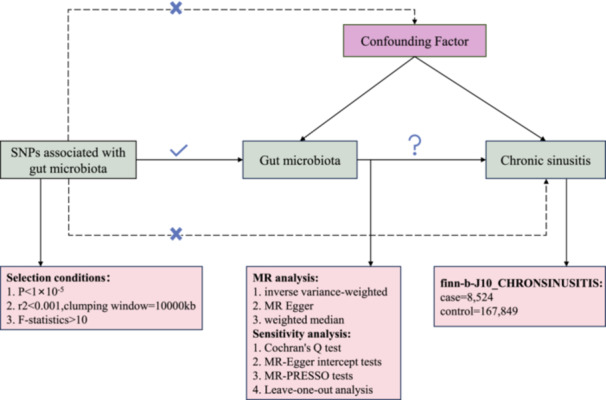
Flowchart of this study. MR‐PRESSO, Mendelian Randomization Pleiotropy RESidual Sum and Outlier; SNP, single‐nucleotide polymorphism.

We employed a two‐sample bidirectional MR design to investigate potential causal effects between gut microbiota and chronic sinusitis. In the forward MR analysis, the exposure variable was considered as the gut microbiota, while the outcome was defined as the chronic sinusitis phenotype. Conversely, in the reverse MR analysis, chronic sinusitis was treated as the exposure variable, and gut microbiota was regarded as the outcome. Through MR modeling with two independent samples, we aimed to explore the presence of bidirectional causal relationships between gut microbiota and chronic sinusitis. Three core assumptions must be met for genetic variation^21^: (1) a significant association between genetic variation and the exposure; (2) genetic variation remains unaffected by confounding factors that may influence both the exposure and outcome variables; and (3) genetic variation influences the outcome through the exposure (Figure 1).

### 2.2 Data sources

The summary statistics of the whole‐genome association study of gut microbiota were derived from the MiBioGen consortium.^22^ This study included 18,340 participants from 24 cohorts, with the majority of participants having European ancestry (*n* = 13,266). They sequenced three different variable regions of the 16S rRNA gene (V1–V2, V3–V4, and V4) and used read‐based taxonomy with the latest reference database (SILVA) for classification. Specifically, they first diluted the samples to 10,000 reads and then employed the RDP classifier (v.2.12) to assign the reads to the reference database, setting a posterior probability of .8 as the cutoff for each read to be classified into a taxonomic interval. They subsequently tracked the posterior cutoff probabilities at each taxonomic level. Subsequently, they conducted microbial quantitative trait loci (mbQTL) mapping analysis, identifying host genetic variations associated with gut microbiota abundance. The researchers analyzed 211 taxonomic groups, including nine phyla, 16 classes, 20 orders, 35 families, and 131 genera.^23^ After excluding unknown taxa, a total of 196 gut microbiota data were included, encompassing nine phyla, 16 classes, 20 orders, 32 families, and 119 genera. The GWAS data for chronic sinusitis were obtained from a large‐scale meta‐analysis publicly available in the Finnish database. This study investigated 8524 cases and 167,849 controls of European populations, involving 16,380,288 single‐nucleotide polymorphisms (SNPs).

### 2.3 Instrumental variables

To select appropriate instrumental variables (IVs) for the gut microbiota, we followed the subsequent steps: Firstly, we established the significance threshold for inclusion of gut microbiota‐related SNPs as *p* < 1 × 10^−5^. Secondly, to ensure independence among the IVs used for the gut microbiota, we set a linkage disequilibrium (LD) threshold for clustering SNP data at *r* ^2^ < .001, with a clustering window size of 10,000 kb. Finally, we calculated the *F* value statistic of the IVs to detect any bias resulting from weak instrumental variables. An *F* value exceeding 10 indicates the absence of bias from weak instrumental variables. Consequently, to mitigate potential biases stemming from weak instruments, we excluded IVs with an *F* value below 10. The *F* value is derived from the formula *R* ^2^(*n*− *k* − 1)/*k*(1 − *R* ^2^), with n denoting the sample size, *k* representing the number of included IVs, and *R* ^2^ indicating the proportion of exposure variance explained by the selected SNPs.

### 2.4 Statistical analysis

The potential impact of gut microbiota features on chronic sinusitis was assessed using multiple statistical models, including inverse variance‐weighted (IVW), MR Egger regression, and weighted median. We used the IVW method as the primary statistical approach, which involves a weighted linear regression of the Wald values for each SNP to obtain an overall estimate. The IVW method, when operating under conditions of homogeneity and absence of horizontal pleiotropy, is considered the most dependable approach since it omits the intercept term in regression analysis.^24^ In comparison to IVW, MR Egger allows for the presence of pleiotropy in over 50% of the IVs and can detect horizontal pleiotropy based on the intercept with the *y* axis.^25^ The weighted median method consolidates data from multiple genetic variants to derive a single causal estimate, ensuring consistent estimates if at least half of the weights are derived from valid IVs. Sensitivity analyses were performed to evaluate the robustness and stability of the obtained results.^26^ To assess potential horizontal pleiotropy, MR Egger intercept tests were conducted. To detect and address any instances of horizontal pleiotropy or outliers, the MR‐PRESSO method was utilized, incorporating Mendelian randomization residual and outlier analysis.^27^ Furthermore, Cochran's *Q* test statistic was utilized to examine heterogeneity among all SNPs. Leave‐one‐out analyses were conducted, sequentially eliminating each SNP to detect potential heterogeneity and evaluate the impact of individual SNPs on the causal association effect. Lastly, reverse MR analysis was conducted on chronic sinusitis and gut microbiota, using methods and settings consistent with forward MR analysis.

To enhance the accuracy of causal effect estimates, we incorporated Bonferroni correction to establish the significance of multiple tests at each feature level (*p* < .05/*n*, where *n* represents the total number of bacterial taxa included within each feature level). Therefore, the significance levels for phylum, class, order, family, and genus were .0055, .0031, .0025, .0015, and .0004, respectively. Statistical analyses were conducted in R software (version 4.3.1) using the “TwoSampleMR” and “MR‐PRESSO” packages.

## 3 RESULTS

### 3.1 SNP selection

After a series of quality control steps, a total of 2086 SNPs related to the gut microbiota were included in the analysis. Specifically, 112, 178, 215, 349, and 1232 SNPs associated with the gut microbiota were identified at the levels of phylum, class, order, family, and genus, respectively. Each result data set did not contain a microbiota group that only consisted of one SNP. Furthermore, all *F* statistic values for the independent variables exceeded 10, indicating no evidence of weak bias.

### 3.2 Causal effects of gut microbiota on the development of CRS

**Figure 2:**
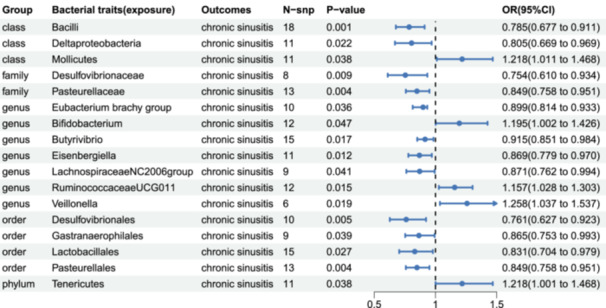
Inverse variance‐weighted forest plot results in forward Mendelian randomization analysis. CI, confidence interval; OR, odds ratio; snp, single‐nucleotide polymorphism.

We employed MR analysis to assess the potential causal relationship between 196 gut microbiota and chronic sinusitis, calculating the odds ratio (OR) and 95% confidence intervals (95% CIs). The results of the IVW method showed that a total of 17 bacterial groups were significantly associated with chronic sinusitis (*p* < .05), including one phylum, three classes, four orders, two families, and seven genera (Figure 2). At the phylum level, the *phylum Tenericutes* (OR: 1.218, 95% CI: 1.011–1.468, *p* = .038) was positively associated with the risk of chronic sinusitis. At the class level, *class Bacilli* (OR: 0.785, 95% CI: 0.677–0.911, *p* = .001) and *class Deltaproteobacteria* (OR: 0.8055, 95% CI: 0.669–0.969, *p* = .022) were protective factors against chronic sinusitis, while *class Mollicutes* (OR: 1.218, 95% CI: 1.011–1.468, *p* = .038) was a risk factor. At the order level, *order Desulfovibrionales* (OR: 0.761, 95% CI: 0.627–0.923, *p* = .005), *order Gastranaerophilales* (OR: 0.865, 95% CI: 0.753–0.993, *p* = .039), *order Lactobacillales* (OR: 0.831, 95% CI: 0.704–0.979, *p* = .027), and *order Pasteurellales* (OR: 0.849, 95% CI: 0.758–0.951, *p* = .004) reduced the risk of chronic sinusitis. At the family level, *family Desulfovibrionaceae* (OR: 0.754, 95% CI: 0.610–0.934, *p* = .009) and *family Pasteurellaceae* (OR: 0.849, 95% CI: 0.758–0.951, *p* = .004) decreased the risk of chronic sinusitis. At the genus level, *genus Eubacteriumbrachy group* (OR: 0.899, 95% CI: 0.814–0.993, *p* = .036), *genus Butyrivibrio* (OR: 0.915, 95% CI: 0.851–0.984, *p* = .017), *genus Eisenbergiella* (OR: 0.869, 95% CI: 0.779–0.970, *p* = .012), and *genus LachnospiraceaeNC2004 group* (OR: 0.871, 95% CI: 0.762–0.994, *p* = .041) were protective factors against chronic sinusitis, while *genus Bifidobacterium* (OR: 1.195, 95% CI: 1.002–1.426, *p* = .047), *genus RuminococcaceaeUCG009* (OR: 1.157, 95% CI: 1.028–1.303, *p* = .015), and *genus Veillonella* (OR: 1.258, 95% CI: 1.037–1.527, *p* = .019) were risk factors for chronic sinusitis. However, after Bonferroni multiple correction, only *class Bacilli* (*p* < .0055) maintained a significant causal relationship with chronic sinusitis and exhibited a negative relationship, while the *order Lactobacillales*, which belongs to class Bacilli, did not pass the multiple corrections.

Based on Cochran's *Q* test, there was no indication of heterogeneity among the specific gut microbiota linked to chronic sinusitis (*p* > .05). MR Egger regression intercept analysis did not demonstrate significant horizontal pleiotropy. In addition, MR‐PRESSO analysis revealed that there was no horizontal pleiotropy among the 17 aforementioned gut microbiota and chronic sinusitis (Table 1). In addition, we did not observe any abnormal IVs for these microbial groups in the leave‐one‐out analysis. Therefore, there is not enough evidence to prove the existence of horizontal pleiotropy between these gut microbiota groups and chronic sinusitis, and our results are stable.

**Table 1:** Sensitivity analysis results for forward MR analysis.

Group	Bacterial traits (exposure)	Outcomes	Cochran's *Q* statistic	Heterogeneity *p* value	MR‐Egger intercept	Intercept *p* value	MR‐PRESSO global test *p* value
Class	*Bacilli*	Chronic sinusitis	17.982	.389	−0.025	.112	0.412
Class	*Deltaproteobacteria*	Chronic sinusitis	8.730	.557	−0.005	.875	0.575
Class	*Mollicutes*	Chronic sinusitis	14.755	.141	0.026	.372	0.176
Family	*Desulfovibrionaceae*	Chronic sinusitis	7.072	.421	−0.007	.834	0.473
Family	*Pasteurellaceae*	Chronic sinusitis	7.574	.817	0.018	.222	0.797
Genus	*Eubacteriumbrachygroup*	Chronic sinusitis	7.024	.634	0.023	.383	0.654
Genus	*Bifidobacterium*	Chronic sinusitis	16.925	.110	0.009	.635	0.095
Genus	*Butyrivibrio*	Chronic sinusitis	14.523	.411	−0.021	.401	0.431
Genus	*Eisenbergiella*	Chronic sinusitis	9.264	.507	0.073	.127	0.542
Genus	*LachnospiraceaeNC2004group*	Chronic sinusitis	8.655	.372	0.049	.148	0.401
Genus	*RuminococcaceaeUCG009*	Chronic sinusitis	9.033	.618	0.011	.625	0.660
Genus	*Veillonella*	Chronic sinusitis	0.635	.986	−0.039	.537	0.987
Order	*Desulfovibrionales*	Chronic sinusitis	7.196	.616	−0.005	.869	0.651
Order	*Gastranaerophilales*	Chronic sinusitis	9.824	.277	0.034	.156	0.287
Order	*Lactobacillales*	Chronic sinusitis	14.794	.392	−0.011	.507	0.411
Order	*Pasteurellales*	Chronic sinusitis	7.574	.817	0.018	.222	0.797
Phylum	*Tenericutes*	Chronic sinusitis	14.755	.141	0.026	.372	0.172

Abbreviation: MR‐PRESSO, Mendelian Randomization Pleiotropy RESidual Sum and Outlier.

In the reverse MR analysis, we found a significant association between chronic sinusitis and five types of intestinal microbiota (*p* < .05) (Table 2). However, there is no significant causal relationship between chronic sinusitis and the aforementioned 17 types of intestinal microbiota. After Bonferroni multiple corrections, the relationship between chronic sinusitis and these five types of intestinal microbiota is no longer significant. The Cochran's *Q* statistics did not reveal significant heterogeneity (*p* > .05). Moreover, both the MR‐Egger intercept test and the MR‐PRESSO test indicated the absence of horizontal pleiotropy (Table 3).

**Table 2:** Results between chronic sinusitis and five significant gut microbiota in reverse MR analysis.

Group	Exposure	Bacterial traits (outcome)	*N*‐snp	Method	SE	OR (95% CI)	*p* Value
Genus	Chronic sinusitis	*Ruminococcaceae UCG013*	5	MR Egger	0.151	0.731 (0.543–0.985)	.131
Genus	Chronic sinusitis	*Ruminococcaceae UCG013*	5	Weighted median	0.059	0.886 (0.88–0.996)	.043
Genus	Chronic sinusitis	*Ruminococcaceae UCG013*	5	IVW	0.044	0.891 (0.816–0.972)	.009
Genus	Chronic sinusitis	*Parabacteroides*	5	MR Egger	0.162	0.964 (0.701–1.325)	.837
Genus	Chronic sinusitis	*Parabacteroides*	5	Weighted median	0.059	0.907 (0.807–1.020)	.104
Genus	Chronic sinusitis	*Parabacteroides*	5	IVW	0.044	0.913 (0.838–0.996)	.040
Genus	Chronic sinusitis	*Coprococcus3*	5	MR Egger	0.157	1.051 (0.772–1.432)	.770
Genus	Chronic sinusitis	*Coprococcus3*	5	Weighted median	0.058	0.907 (0.809–1.017)	.096
Genus	Chronic sinusitis	*Coprococcus3*	5	IVW	0.045	0.910 (0.831–0.995)	.040
Genus	Chronic sinusitis	*Candidatus Soleaferrea*	5	MR Egger	0.246	1.628 (1.004–2.641)	.142
Genus	Chronic sinusitis	*Candidatus Soleaferrea*	5	Weighted median	0.092	1.212 (1.012–1.452)	.036
Genus	Chronic sinusitis	*Candidatus Soleaferrea*	5	IVW	0.072	1.223 (1.060–1.408)	.005
Family	Chronic sinusitis	*Lachnospiraceae*	5	MR Egger	0.147	0.870 (0.651–1.162)	.415
Family	Chronic sinusitis	*Lachnospiraceae*	5	Weighted median	0.053	0.909 (0.818–1.010)	.077
Family	Chronic sinusitis	*Lachnospiraceae*	5	IVW	0.042	0.903 (0.831–0.982)	.018

Abbreviations: CI, confidence interval; IVW, inverse variance‐weighted; MR, Mendelian randomization; OR, odds ratio; SE, standard error; snp, single‐nucleotide polymorphism.

**Table 3:** Sensitivity analysis results for reverse MR analysis.

Group	Exposure	Bacterial traits (outcome)	Cochran's *Q* statistic	Heterogeneity *p* value	MR‐Egger intercept	Intercept *p* value	MR‐PRESSO global test *p* value
Genus	Chronic sinusitis	*Ruminococcaceae UCG013*	4.040	.401	0.027	.267	.479
Genus	Chronic sinusitis	*Parabacteroides*	3.551	.470	−0.007	.750	.537
Genus	Chronic sinusitis	*Coprococcus3*	1.604	.807	−0.019	.409	.818
Genus	Chronic sinusitis	*Candidatus Soleaferrea*	1.816	.769	−0.039	.311	.783
Family	Chronic sinusitis	*Lachnospiraceae*	1.636	.802	0.005	.805	.820

Abbreviation: MR‐PRESSO, Mendelian Randomization Pleiotropy RESidual Sum and Outlier.

## 4 DISCUSSION

The gut microbiota and respiratory microbiota are known to be interconnected, as previous studies have shown a high degree of similarity in microbial colonization between these two sites.^28^, ^29^ They share similar microbial communities primarily composed of Firmicutes and Bacteroidetes phyla.^30^, ^31^ Moreover, there is ongoing interaction between the microbiota of these two sites. Research has found that respiratory microbiota development can be influenced by microbial respiration in the gut,^32^ and many bacterial species present in the gut also exist in the respiratory tract.^33^ Therefore, respiratory infections and related diseases may be associated with dysbiosis in the gut microbiota. Animal experiments have observed alterations in the gut microbiota of mice following respiratory viral infections.^34^

Our study has confirmed that the Bacilli class is a protective factor for chronic sinusitis, which may be related to its antibacterial properties. The microbiota can enhance the function of the intestinal mucosal barrier, preventing the colonization of pathogenic bacteria and thus exerting a protective anti‐inflammatory effect.^35^ Furthermore, the gut microbiota produces enzymes, vitamins, and synthesizes short‐chain fatty acids through diet to stimulate the production of antibacterial factors in the intestinal epithelium.^36^ Previous research has shown that metabolites of Bacilli include acetates, propionates, and butyrate, among which butyrate play a significant role in improving mucosal inflammation, oxidative status, and enhancing intestinal mucosal barrier function.^37^ Previous animal experiments have shown that butyrate can reduce inflammation in mice implanted with polyether polyurethane sponges, characterized by reduced neutrophil infiltration and decreased levels of tumor necrosis factor‐α, interleukin‐10, and transforming growth factor‐β1.^38^ Therefore, the protective effect of Bacilli on chronic sinusitis may be mediated through its metabolites. On the other hand, the protective effect of Bacilli on chronic sinusitis may be achieved through immunological processes. The gut microbiota and their metabolites are involved in mucosal immune responses (including gastrointestinal mucosa and respiratory mucosa).^39^ They signal through the gut nervous system or hormones to evolve B lymphocytes into plasma cells that spread throughout the body, producing secretory immunoglobulin A (Sig A) involved in innate immune processes, thereby preventing pathogenic bacteria and viruses from adhering to the epithelium.^40^, ^41^ Their immune activation is mediated by pattern recognition receptors (PRRs), particularly Toll‐like receptors (TLRs).^42^ Toll‐like receptor 4 (TLR4) present in immune cells (macrophages, mast cells, lymphocytes, etc.) activates these immune cells by binding with LPS, thereby promoting the production of anti‐inflammatory factors.^43^

Our study has certain limitations. Firstly, the participants in the GWAS samples for exposure and outcome were predominantly of European descent, so further validation is needed to determine if the results are applicable to other populations. Secondly, due to the lack of more detailed subcategorization of chronic sinusitis data, we did not perform further subgroup analysis, which limits our ability to obtain more specific causal relationships. Furthermore, caution is advised when interpreting significant results, as we found no microbial taxa passing correction through the Benjamini–Hochberg method for controlling the false discovery rate (FDR) in our MR analysis results. Lastly, the GWAS data on gut microbiota used in this study is limited, with many not included. In the future, incorporating additional advanced metagenomic sequencing analyses will make the results more specific and accurate. Although we have identified a potential association between gut microbiota and chronic sinusitis, a more precise relationship needs to be established through additional evidence such as cohort studies and clinical trials, particularly regarding the mechanisms involved.

## 5 CONCLUSION

Based on two samples of Mendelian randomization studies, we have successfully identified a plausible causal relationship between gut microbiota and chronic sinusitis. Our research findings hold potential significance for the clinical prevention and management of chronic sinusitis.

## AUTHOR CONTRIBUTIONS

**Kunlin Pu:** Conceptualization; writing—original draft; formal analysis. **Zhipeng Zhang:** Data curation; visualization. **Li Li:** Conceptualization.

## Data Availability

This study analyzed publicly available data sets. Data on chronic sinusitis are available at the following website: IEU OpenGWAS project (mrcieu.ac.uk). Summary statistics on the gut microbiota are available at (https://mibiogen.gcc.rug.nl/).
